# BOLSTERING SENSE OF PURPOSE FOR INDIVIDUALS LIVING WITH EARLY DEMENTIA: AN APPRECIATIVE INQUIRY APPROACH

**DOI:** 10.1093/geroni/igae098.0338

**Published:** 2024-12-31

**Authors:** Emily Mroz, Joan Monin, Kate Keefe, Erica DeFrancesco, Heidi Gil

**Affiliations:** Emory University, Decatur, Georgia, United States; Yale School of Public Health, New Haven, Connecticut, United States; LiveWell Institute, Southington, Connecticut, United States; LiveWell Institute, Southington, Connecticut, United States; LiveWell Institute, Southington, Connecticut, United States

## Abstract

Maintenance of a sense of purpose is a hallmark of wellbeing. Individuals consistently report a diminished sense of purpose in the wake of a diagnosis of Alzheimer’s disease or related dementias (ADRD). One recently-proposed hypothesis (Sutin et al., 2023) is that this trend is a consequence of early, ADRD-related neurodegeneration, implying that adults living with early ADRD may be less cognitively able to pursue purpose. In contrast, our team hypothesizes that these declines are the result of a lack of evidence-based practices in clinical and community settings focused on bolstering purpose following this life-changing diagnosis. Our team pursued Stage 0 research given the lack of existing interventions. In partnership with people (N = 17) living with mild-moderate ADRD recruited from the Empowering Partnership Network, we completed a Design Thinking workshop using an Appreciative Inquiry method (Stowell, 2013). Workshop insights were recorded, and the first author completed qualitative thick descriptions to synthesize insights. Findings establish that, with the availability of support, adults with mild-moderate ADRD can generate meaningful purposes. Findings also described how the ADRD diagnosis journey can undermine purpose and suggest opportunities for multicomponent, community-based interventions to bolster purpose following diagnosis. This presentation will end with recommendations for partnering with people living with ADRD and applying the Appreciative Inquiry method at Stage 0. We will also discuss how our findings help to re-define living well with purpose and ADRD, and to refine the Healthy Lifestyle Course, a novel community-based resource that shows promise for bolstering purpose in this population.

